# Co-expression based cancer staging and application

**DOI:** 10.1038/s41598-020-67476-7

**Published:** 2020-06-30

**Authors:** Xiangchun Yu, Sha Cao, Yi Zhou, Zhezhou Yu, Ying Xu

**Affiliations:** 1 Cancer Systems Biology Center, The China-Japan Union Hospital, Jilin University, Changchun, China; 2 College of Computer Science and Technology, Jilin University, Changchun, China; 3 Computational Systems Biology Lab, Department of Biochemistry and Molecular Biology and Institute of Bioinformatics, University of Georgia, Athens, USA; 4 Department of Biostatistics, Indiana University School of Medicine, Indianapolis, USA; 5 School of Information Engineering, Jiangxi University of Science and Technology, Ganzhou, China

**Keywords:** Cancer genomics, Cancer models

## Abstract

A novel method is developed for predicting the stage of a cancer tissue based on the consistency level between the co-expression patterns in the given sample and samples in a specific stage. The basis for the prediction method is that cancer samples of the same stage share common functionalities as reflected by the co-expression patterns, which are distinct from samples in the other stages. Test results reveal that our prediction results are as good or potentially better than manually annotated stages by cancer pathologists. This new co-expression-based capability enables us to study how functionalities of cancer samples change as they evolve from early to the advanced stage. New and exciting results are discovered through such functional analyses, which offer new insights about what functions tend to be lost at what stage compared to the control tissues and similarly what new functions emerge as a cancer advances. To the best of our knowledge, this new capability represents the first computational method for accurately staging a cancer sample. The R source code used in this study is available at GitHub (https://github.com/yxchspring/CECS).

## Introduction

We present a computational approach to stage accurately cancer tissues based on their RNA-seq data. The stage of a cancer is a key parameter for clinically characterizing the cancer. As a cancer advances, the disease generally evolves from a localized issue to a whole-body problem^1–3^, not just in term of whether a cancer is metastasized or not, as cancer tends to persistently release certain molecules such as protons, cytokines and polyamines^4–6^ as well as “consume” certain molecules like sodium and iron, leading to substantial alterations of their blood concentrations over time. For some molecular species, such changes will trigger highly damaging responses by different organs throughout the body. Cachexia, i.e., loss of muscle cells throughout the body, is one consequence of such responses towards the advanced stage of a cancer^7–10^ Intracellularly, considerable changes take place in metabolisms as a cancer evolves, giving rise to gradual and extensive metabolic reprogramming in cancer^11–14^. Hence, cancers detected at different stages require distinct treatment plans. Therefore, accurate staging of a cancer is vitally important to the cancer patient and his/her physician.

Somewhat surprisingly, the clinical practice of cancer staging has not changed much in the past 40 years^15–17^ as it is still done predominantly based on the morphology and the size of a cancer tissue, examined manually by cancer pathologists under microscope, assisted by limited protein biomarkers. One would intuitively expect that cancer staging nowadays should have been done in a more objective manner based on molecular data, knowing that cancer tissue omic data, particularly gene-expression data are easily obtainable in a financially viable manner. However, the reality is: while gene-expression data represent the easiest to get and the most informative omic data for studying cancer tissues, they have not been widely used for cancer staging outside of laboratory studies^18–20^. Published work is mostly on transcriptomic biomarkers for cancer prognostic prediction^21–27^ rather than cancer staging.

A key challenge in achieving this goal comes from the reality that scientists have yet to identify genes whose (differential) expression patterns in cancer vs. controls are specifically associated with individual stages of a cancer type, and hence can be used for cancer-stage prediction. Our own analyses have discovered that co-expression patterns are considerably more informative than differential expressions of individual genes for cancer staging. Here we present a co-expression based cancer staging method. To the best of our knowledge, there are no published studies that predict cancer stages using co-expression patterns of cancer tissues.

A technical challenge in applying co-expression data for cancer staging is: how to derive co-expression information of genes in individual tissue samples since it generally requires multiple samples to infer such information while cancer staging needs to be done on individual tissues. Fortunately, Chen and co-workers have recently published a statistical method for inference of co-expressed genes in a single sample through comparing the co-expressed genes in a set of reference samples and those in the reference set plus the current sample^28^. Specifically, the approach assesses if the co-expression patterns among the reference samples are enhanced or weakened by including the sample into the reference set, namely an expanded set. A pair of genes in the new sample is considered as having the same co-expression pattern in the reference set and the expanded set if its co-expression level in the latter is not statistically lower than in the former. Hence, when applied to all gene pairs, a set of co-expressed genes can be derived for the given sample with respect to the reference set. This method has been applied to solving a variety of co-expression analysis problems and found to be highly effective^28^.

We have adapted and applied this approach to cancer tissue staging. Specifically, we assume that some samples for each stage of a cancer type are available, along with their genome-scale transcriptomic data, from which co-expression patterns can be derived reliably for each stage of the cancer type. Then a new sample is assigned to a stage if the sample’s co-expression pattern is most consistent with the co-expression patterns of the stage of the reference samples within a specified level of difference. We have applied this staging approach to eight cancer types in the TCGA database for stage prediction, representing all the cancer types that has at least ten cancer samples in each of the four stages. The consistency levels range from 71 to 95% across the eight cancer types we studied. The reason we have applied our method only to the TCGA data is that the data are collected from cancer tissue samples, rather than cell lines^29,30^, with the highest data quality compared to other databases.

An important application of this methodology is to elucidate the functional differences between cancer samples at different stages, hence providing important and useful information regarding cancer evolution from early to the advanced stage. To do this, we have developed a new method for assessing the statistical significance of pathways enriched by a set of gene pairs rather than a set of genes as commonly done. By applying this method, we have examined what normal functions tend to disappear at what stage and what new functions may emerge at what stage of a cancer type. This functional analysis results have revealed novel understanding about cancer evolution, hence providing concrete examples for a profound postulation made by Otto Warburg 50 ago: “the highly differentiated cells are now transformed into fermenting anaerobes, which have lost all their body functions and retain only the now useless function of growth”^31–33^.

## Results

Gene expression data of eight cancer types, namely BRCA, COAD, HNSC, KIRC, KIRP, LUAD, STAD and THCA, are extracted from the TCGA database. Our cancer-stage prediction is conducted and assessed on these samples. The detailed information about these cancer data are given in the Methods section.

### Identification of co-expressed genes

For each cancer type, *edgeR* in the R package is used to identify the differentially expressed genes (DEGs) using $|\mathrm{l}\mathrm{o}\mathrm{g}(\mathrm{F}\mathrm{C})|>2.5$ and *p* value < 0.05 as the cutoffs. Pearson correlation coefficient (PCC) is used to calculate the co-expression level between two genes. A pair of genes (x, y) is deemed to be co-expressed (CEGs) if $\left|\mathrm{P}\mathrm{C}\mathrm{C}\left(\mathrm{x},\mathrm{y}\right)\right|>2.5$ with *p* value < 0.05 (see Methods). Table 1 summarizes the numbers of DEGs and CEGs for each cancer type at each stage.

**Table 1:** The numbers of DEGs and CEGs in each of the eight cancer types.

Stage	#DEGs and #CEGs		BRCA	COAD	HNSC	KIRC	KIRP	LUAD	STAD	THCA
Stage 1 vs. control	#DEGs	Up	1,255	1,718	347	1,110	640	2,004	673	893
		Down	512	729	975	594	735	129	670	228
	#CEGs	Up	61,638	385,089	1,428	56,850	1,113	130,324	3,717	3,651
		Down	1,690	11,763	26,804	920	2,564	121	15,338	600
Stage 2 vs. control	#DEGs	Up	1,564	1,581	547	1,399	700	1,268	662	662
		Down	527	681	943	560	784	190	713	488
	#CEGs	Up	14,410	143,619	634	62,299	14,452	3,102	1,892	7,835
		Down	981	6,472	25,765	9,450	13,173	258	9,888	9,125
Stage 3 vs. control	#DEGs	Up	1,040	1,607	597	1,159	955	1,269	597	903
		Down	553	588	949	739	640	217	744	289
	#CEGs	Up	3,925	104,838	1,308	8,426	5,864	2,847	572	5,109
		Down	1,912	6,200	16,024	2,306	784	1,007	7,295	2,074
Stage 4 vs. control	#DEGs	Up	818	1,035	798	1,325	657	1,097	504	932
		Down	721	685	872	727	727	156	939	535
	#CEGs	Up	7,386	4,597	542	18,161	9,068	14,427	952	3,449
		Down	15,550	8,372	15,892	5,343	24,119	923	13,429	3,142

### An algorithm for representing cancer samples as co-expression networks

We have developed an algorithm for representing the gene-expression data of cancer tissue samples of a given cancer type as four stage-specific co-expression networks, one for each stage, and their perturbed networks when a new sample is added to the sample set of each stage. The level of perturbation due to inclusion of the new sample to each of the four co-expression networks, in general, will be significantly different between the network where the new sample intrinsically belongs and the three other networks. This serves as the basis of our cancer staging algorithm.

A co-expression network is built over samples in each stage of a given cancer type, consisting of only gene pairs that are highly co-expressed, where each gene pair is represented as an edge connecting two nodes denoting the two genes. When a new sample is added to the sample set of each stage, the co-expression levels of some gene pairs may change. Chen and co-authors have made the following observation^28^: if two genes are co-expressed over a sample set, then adding a new sample to the set should not change their co-expression level significantly if their expression levels in the new sample are linearly consistent with those in the sample set; otherwise the co-expression level will decrease or remain at a low level. In addition, we have noted that cancer samples in the same stage tend to have a large collection of stage-specific co-expressed genes, used to execute the biological functions specific to the stage. By integrating these two insights, we have the following key observation: **for a given co-expression network of a specific stage, adding a new sample that “intrinsically” belongs to the stage should not alter significantly the structure of the co-expression network**; in contrast, when a sample is added to the sample set of a different stage, it will affect the co-expression levels of some gene pairs, hence altering the structure of the co-expression network. Our algorithm follows.

**Step 1:** *Identification of DEGs for co-expression analyses*. To ensure that the numbers of DEGs are approximately the same across different stages to avoid sample-size related bias, we have selected *n* DEGs with the largest variance for each stage, where *n* is the smallest number of DEGs in a stage across the four for the given cancer type.

**Step 2**: *Construction of co-expression networks.* Samples of each stage are divided into three groups: 30% as the reference, 40% for training, and 30% for testing. A co-expression network is constructed over the reference set for each of the four stages: each DEG is defined as a node and a pair of co-expressed DEGs above a PCC-based threshold (see METHODS) as an edge linking the two genes.

**Step 3:** *Construction of a perturbed network over each sample set plus a new sample.* For each co-expression network N built at Step 2 and a new sample s, calculate the PCC value for each co-expressed gene pair in N over the expanded sample set. If the relationship between the new PCC and the threshold is reversed compared to the original PCC, remove it from N if PCC > threshold; and otherwise add the edge to N.

**Step 4**: *Data preparation for cancer-stage classifier training*. For each new sample considered for cancer staging, represent each of its four perturbed networks as a one-dimensional vector: each pair of co-expressed genes in a co-expression network is given a fixed location in the vector, containing the PCC value or a 0.0 if the gene pair is removed in the perturbed network, hence allowing direct comparisons among such PCC-based vectors.

**Figure 1:**
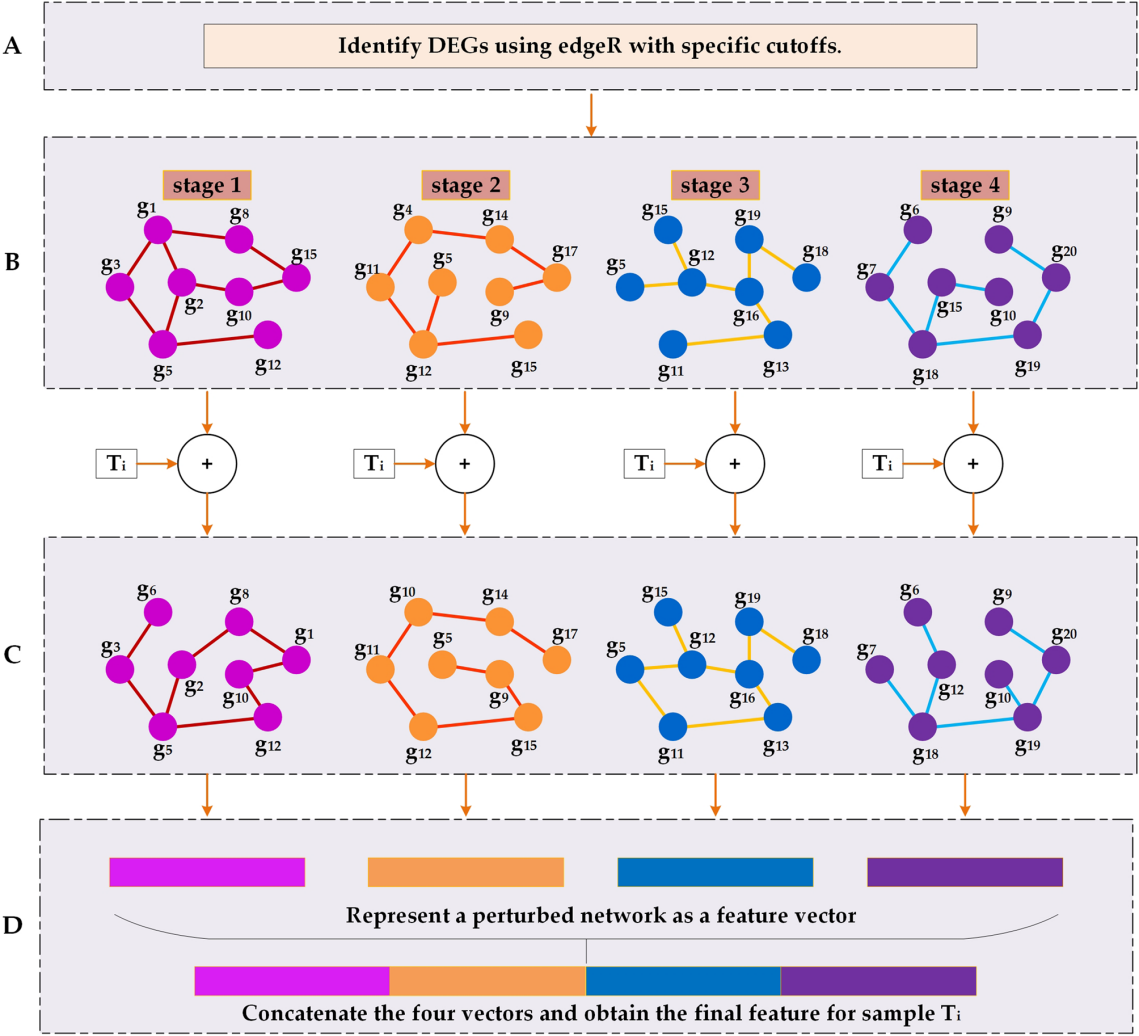
An illustration of our algorithm. (**A**) Identification of DEGs between cancer versus control tissues at each stage. (**B**) Construction of co-expression networks for samples in each of the four stages with the DEGs obtained from step A. (**C**) Construction of perturbed networks over samples in each stage plus a new sample denoted by *T*_*i*_. (**D**) Representation of each perturbed network as a feature vector needed for training, giving rise to four feature vectors concatenated into a long vector, which will be fed into a trainer as the feature vector for sample *T*_*i*_.

The detailed process of our algorithm is shown in Fig. 1.

### A 4-way classifier for cancer staging

A machine learning-based classifier is trained to predict the stage, 1 through 4, for a given cancer sample based on the PCC vectors defined in Sect. 2.1. Intuitively, if a new sample belongs to a specific stage, its perturbed network should be largely the same as the corresponding co-expression network; otherwise, the perturbed network may lose most of the stage-specific co-expressed genes, i.e., edges, from the original co-expression network.

We have used the following six machine-learning methods: Naive Bayes, treebag, C5.0, random forests (RF), random ferns (RFerns), and weighted subspace random forests (WSRF), respectively, to train the classifier.

### Cancer stage prediction

Using the above cancer-staging algorithm, we have predicted stages for all the test samples of the eight cancer types. For each cancer type, we have randomly selected 30% of the samples from each stage and used them to derive the co-expression patterns; 40% for classification model training; and the remaining 30% for testing. Three-fold cross-validation with 100 repeats is used when training a classifier for each of the six machine learning methods. This process is iterated 10 times, and the average of the staging accuracy is used as the final evaluation results.

Table 2 summarizes the prediction results by C5.0, and prediction results by other methods are summarized in Supplementary Tables S1(1–5). We note that most of the machine learning methods give comparable results except for Naive Bayes and random Ferns, whose performances are poorer than the others as detailed in the Table S1.

**Table 2:** Prediction performance of cancer stages using C5.0.

Stage	Measure	BRCA	COAD	HNSC	KIRC	KIRP	LUAD	STAD	THCA
1	Sensitivity	0.7852	0.9227	0.6714	0.9519	0.9353	0.8795	0.24	0.9753
	Specificity	0.983	0.9927	0.8783	0.9737	0.9	0.9227	0.9678	0.9172
2	Sensitivity	0.9409	0.9755	0.51	0.8938	0.6	0.74	0.8	0.9333
	Specificity	0.9737	0.9641	0.9393	0.977	0.9928	0.9377	0.9257	0.9836
3	Sensitivity	0.7932	0.9237	0.4696	0.9639	0.7714	0.6667	0.7955	0.8212
	Specificity	0.9722	0.9817	0.926	0.9933	0.9508	0.9688	0.7983	0.9422
4	Sensitivity	0.72	0.9667	0.8675	0.9292	0.8	0.4714	0.7273	0.4188
	Specificity	0.922	0.9894	0.886	0.9809	0.9465	0.8965	0.889	0.9692
All	Accuracy	0.8768	0.9504	0.7283	0.9452	0.8707	0.7933	0.7078	0.8772
	Kappa	0.7957	0.9293	0.546	0.9175	0.7436	0.6807	0.5696	0.7928

To understand what might be the reasons for the inconsistent predictions by our method compared to the **annotated** stages in TCGA by pathologists, we have examined the prediction results for HNSC and STAD, the two cancer types with the worst overall prediction performance (Table 2). Tables 3 and 4 list, for each stage, the numbers of samples correctly predicted and of predicted to earlier or later stages of HNSC and STAD, respectively.

**Table 3:** The confusion matrix for predicted vs. annotated stage of HNSC.

Predicted/annotated	Stage 1	Stage 2	Stage 3	Stage 4
Stage 1	4	5	2	1
Stage 2	2	14	5	5
Stage 3	1	0	16	9
Stage 4	0	1	0	62

**Table 4:** The confusion matrix for predicted vs. annotated stage of STAD.

Predicted/actual	Stage 1	Stage 2	Stage 3	Stage 4
Stage 1	8	1	2	0
Stage 2	0	19	3	0
Stage 3	4	11	37	2
Stage 4	3	1	2	9

**Figure 2:**
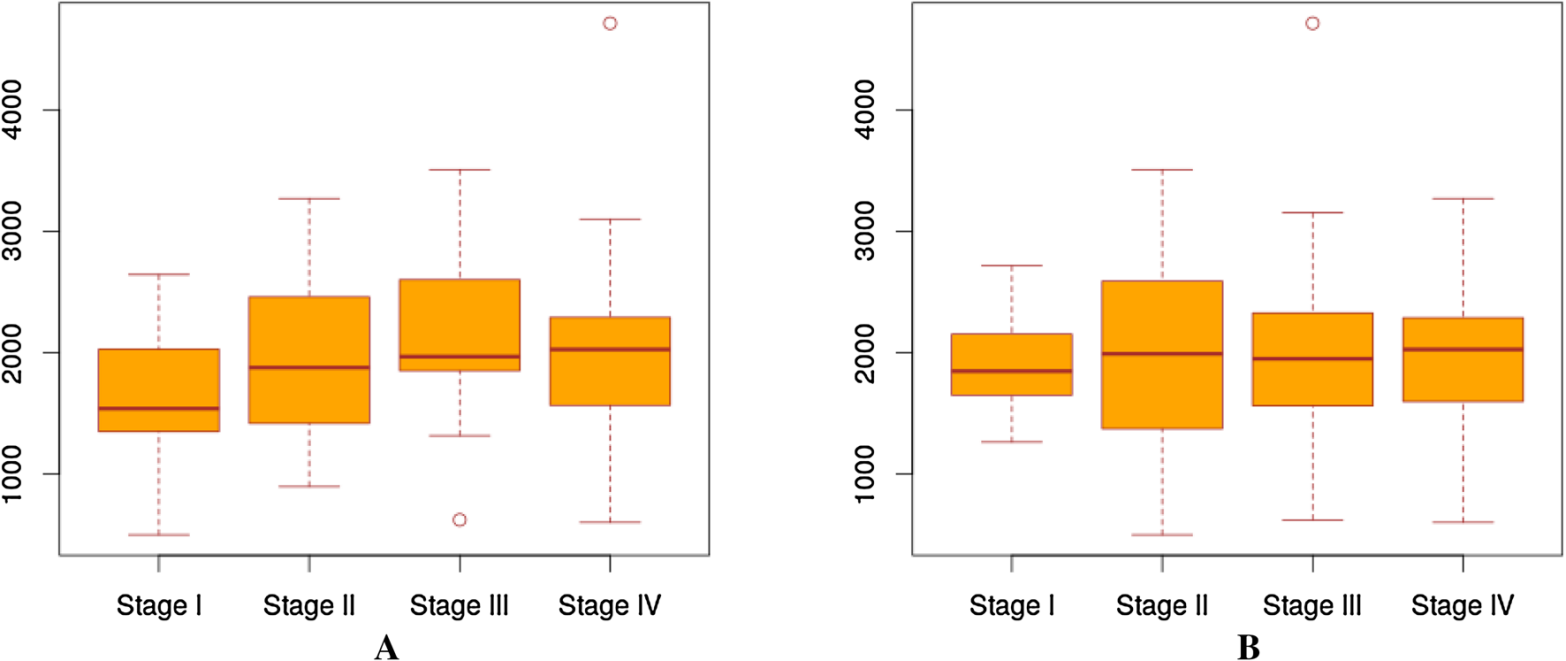
The distributions of the number of DEGs across samples at different “stages” by the manually annotated stages and our predicted stages. (**a**) The distribution of the numbers of DEGs (y-axis) in each annotated stage of HNSC. (**b**) The distribution of the numbers of DEGs in each predicted stage of HNSC.

Since there is no ground truth for the actual stages of the cancer samples under consideration that can be used to assess the quality of the two staging methods, we have compared the distributions of the number of DEGs across samples at different “stages” by the two methods, as shown in Fig. 2. We see from the boxplots that our predicted stages give rise to boxplots with somewhat higher level of regularity compared to that of the annotated stages, hence providing one piece of evidence that our predicted stages, which is based on molecular information, might be more intuitively meaningful.

**Figure 3:**
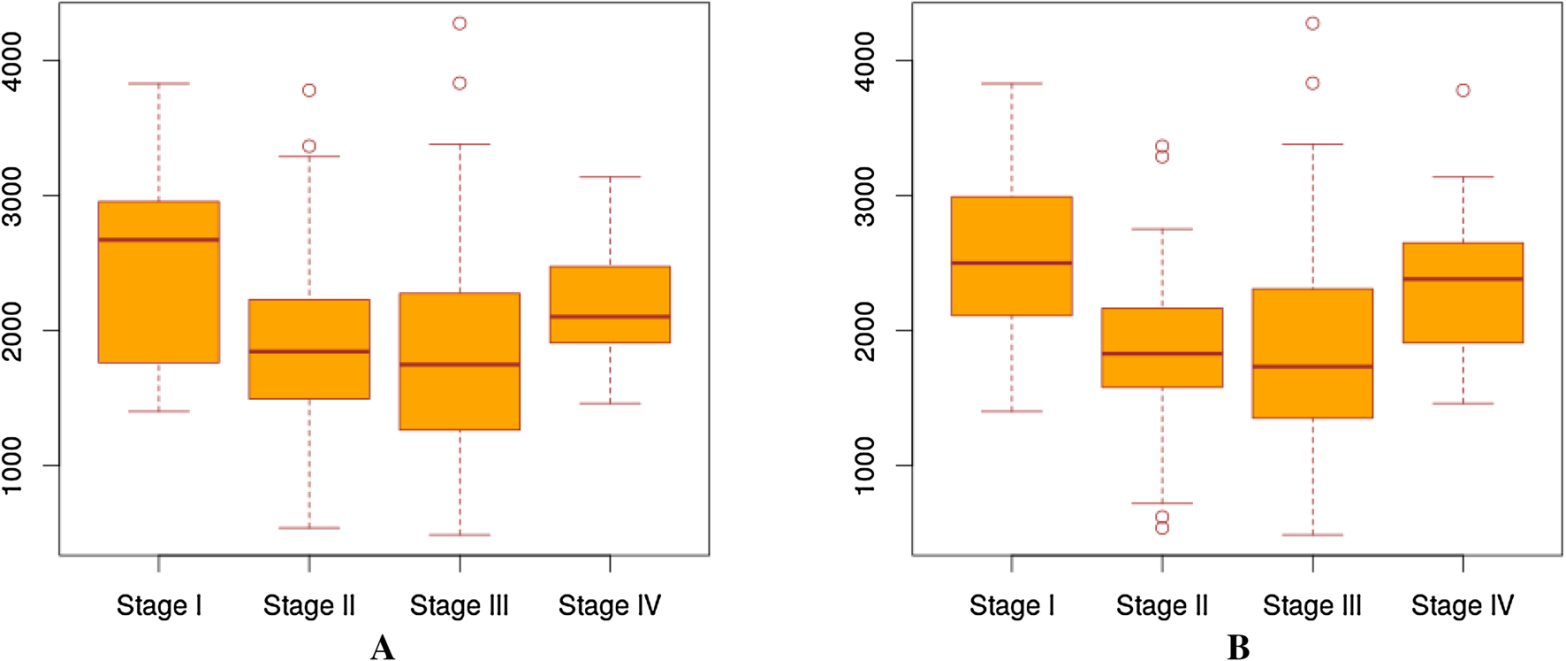
The distributions of the number of DEGs across samples at different “stages” by the manually annotated stages and our predicted stages. (**a**) The distribution of the numbers of DEGs in each annotated stage of STAD. (**b**) The distribution of the numbers of DEGs in each predicted stage of STAD.

Figure 3 shows the similar information for STAD to that in Fig. 2. Analysis results on other cancer types are given in Supplementary Tables S2(1–6) and Figures S1(1–6). Overall, we consider that our predicted stages are probably as scientifically justified as the manually annotated stages by cancer pathologists or better.

### Pathways enriched by co-expressed genes

We have conducted pathway enrichment analyses over co-expressed genes in each stage of each of the eight cancers against the GO Biological Processes using our new scoring scheme (see “Methods”). Table 5 summarizes the numbers of the enriched pathways by co-expressed genes, with the pathway names given in Supplementary Tables S3-1 (controls), S3-2 (up-regulated), and S3-3 (down-regulated), and information about pathways, hence functions, that disappear at each stage as well as new pathways that emerge at each stage in cancer versus controls, hence providing footprint information of cancer evolution.

**Table 5:** The numbers of pathways enriched by co-expressed genes in controls and at each stage.

Stage	#CEPs	BRCA	COAD	HNSC	KIRC	KIRP	LUAD	STAD	THCA
Stage 1 vs. control	Control	223	178	126	195	138	48	84	44
	Up	120	13	74	22	4	46	53	82
	Down	71	102	92	34	66	6	66	2
Stage 2 vs. control	Control	323	150	136	181	109	56	102	137
	Up	166	25	39	135	66	54	18	16
	Down	83	115	104	2	117	5	65	164
Stage 3 vs. control	Control	251	102	111	201	99	53	102	57
	Up	143	25	27	265	81	41	35	85
	Down	73	81	114	17	17	9	74	20
Stage 4 vs. control	Control	326	131	111	226	125	100	205	119
	Up	135	38	27	313	99	110	34	56
	Down	109	70	108	33	66	5	100	58

^#^CEPs is for the number of co-expressed gene pairs; Up is for the number of CEPs by up-regulated genes; and Down is similarly for down-regulated genes.

We have also calculated the numbers of enriched pathways by co-expressed genes in controls, which (I) remain enriched throughout all stages of the cancer samples of each type; and (II) disappear by each stage of cancer samples, which do not appear again in a later stage, and in total for each cancer type. And we have also calculated (III) the number of new pathways that are not present in controls but present in earlier stages (1 and 2) or advanced stages (3 and 4). All these are shown in Table 6 (I), (II) and (III), and the detailed pathways in cancer are listed in Supplementary Tables S4-1, S4-2 and S4-3. From these tables, we conclude:

(i) it is somewhat surprising to see from Table S4-1 that different sets of functions remain unchanged throughout the development of a cancer type across the eight cancer types. For example, for BRCA, it is cell cycle and cell division activities that represent the predominant class of functions that remain unchanged throughout stages 1–4. And this is the only type of cancer with this or similar property. For COAD, it is three classes of functions, namely cellular stress, immune responses and tissue repair that remain unchanged throughout the evolution of the cancer. For HNSC, it is the combination of two functional classes: tissue repair and cellular stress that remain unchanged throughout its evolution. For KIRC, no functional activities remain unchanged throughout its evolution. For KIRP, it is some developmental activities that remain unchanged. For LUAD, it is a few cell division activities that remain unchanged. And for STAD, it is predominantly immune responses that remain changed.
(ii) from Table S4-2, we see the following: (1) pathway disappearance in cancer predominantly take place at stage 1 for six cancer types or stage 4 for two cancer types; and (2) most of the lost pathways tend to be cancer specific or at most shared by 2–3 cancer types except for a few, namely neutral lipid metabolic process (shared by 6 cancer types), triglyceride metabolic process (shared by 5), acylglycerol metabolic process (by 5), response to drug (by 4), regulation of lipid localization (by 4), regulation of hormone levels (by 4), and organic anion transport (by 4), indicating that they may have negative effects on cancer development, hence selected for removal. The detailed list of the lost pathways by multiple cancer types is given in Table S5.
(iii) from Table S4-3, we note that different cancer types tend to have different sets of emerging functions in cancer tissues vs. controls, which generally fall into the following classes: development and proliferation, immune related, stress related, migration related, metabolisms, tissue repair, and neural functions.

**Table 6:** The number of enriched pathways in normal controls.

#CEPs		BRCA	COAD	HNSC	KIRC	KIRP	LUAD	STAD	THCA
Total		442	274	168	355	261	133	264	257
(I)		69	29	78	0	3	5	34	9
(II)	Stage 1	91	78	20	103	68	21	21	16
	Stage 2	64	31	7	7	17	3	20	20
	Stage 3	16	8	1	9	9	0	19	14
	Stage 4	56	11	0	11	9	7	85	40
	Total	227	128	28	130	103	31	145	90
(III)	1–2	106	52	89	29	118	48	50	99
	3,4	166	66	40	163	111	36	69	74

Total on the second row is the number of pathways enriched by CEPs in control samples for each cancer type while Total under (II) is for the number of unique pathways enriched by CEPs across all cancer samples of each type.

For BRCA, two classes of new functions account for the majority of the new functions, hence considered as predominant: development and proliferation and metabolisms in both early (stages 1 and 2) and advanced (stages 3–4) cancers.

For COAD, the two predominant functional classes are development and proliferation and stress related in both early and advanced cancers.

For HNSC, the new functions in early-stage cancer tissues are development and proliferation and immune related; and for the advanced tissues, only the former remains to be predominant.

For KIRC, no single class of functions stands out in the early stage; and immune related and development and proliferation stand out.

For KIRP, development and proliferation and metabolisms stand out in both the early and advanced cancer tissues.

For LUAD, development and proliferation and stress related functions stand out in the early stage; and the latter changes to neural activities in the advanced stage.

For STAD, tissue repair and immune related functions stand out in both early and advanced stages. In addition, development and proliferation become one of three standout functional classes with the other two in the advanced stage cancer tissues.

For THCA, immune and tissue repair stand out in the early stage; and the former changes to development and proliferation in the advanced stage.

Among these functions, development and proliferation related functions become increasingly predominant as a cancer advances from early to the advanced stage for virtually all cancer types. Similarly, the percentages of the following functions also increase as a cancer advances: stress, immune, and migration related.

## Discussion

Our preliminary analyses strongly indicate that differential expressions of individual genes do not have adequate information for accurate cancer staging, and conserved co-expression patterns across cancer samples of the same stage do as we have demonstrated through here. This represents a key technical contribution to the research of cancer biology. We anticipate that a similar technique could be used for various similar problems such as cancer grading, classification of primary cancers that have metastasized vs. that have not.

Our prediction results are generally consistent with those assigned manually by cancer pathologists. In cases where our predictions are inconsistent with the manual annotation, further studies are needed as there are no clear indication of which “predictions” are more accurate between the two, although from one specific angle, our predictions seem to be biologically more meaningful. This should not be surprising since our prediction is based on functional commonalities shared by most of the cancer tissue samples of a specific stage. We anticipate that systematic applications of this new tool could lead to improved and biologically more meaningful staging schemes for different cancer types. For example, by studying how the overall functionality of cancer samples changes as a cancer advances, one could possibly identify key “jumps” in changes in the total functionality, which can be used to distinguish distinct phases of the evolution for specific cancer types, compared to the current staging schemes, which are largely based on sizes and morphology of tumors. Cancer staging based on such molecular functions could lead to improved treatment plans that can target at key functional hubs or weakest points in cancer metabolic networks at distinct phases.

Otto Warburg speculated fifty some years ago about cancer evolution as: “the highly differentiated cells are now transformed into fermenting anaerobes, which have lost all their body functions and retain only the now useless function of growth”^31–33^. Since then, very little has been established regarding what specific functions are lost as a cancer evolves. We consider that a scientific contribution made by this study is: we have provided some information along this direction, although our study is clearly primitive. A further study is planned to elucidate detailed functionalities of cancer at individual stage and of different types. Both functionalities shared by all or most of the cancer types and specific to individual cancer types are of great interests. Our co-expression based functional identification will prove to be a highly effective tool for conducting such studies.

Regarding the predominant new functions in cancer vs. controls as revealed by our analyses, it is understandable why development and proliferation represents a predominant one across a majority of the cancer types under study as cancer proliferation, unlike normal developmental processes, may require segments from multiple developmental programs, which might be activated possibly by different signals for different reasons such as the need for tissue repair, to have the cell-cycle genes activated and form a somewhat coordinated cell cycle process in support of continuous cell proliferation. Other emerging functions, such as immune, tissue repair, metabolisms and/or neural activities, tend to be less conserved across different cancer types. Hence it is natural to ask: are new functions in each cancer type relevant to or even possibly dictate the clinical behaviors of different cancer types such as more vs. less malignant cancers? Clearly, further and more in-depth analyses are clearly needed to address this question.

## Conclusion

A new algorithm for cancer staging is developed based on co-expression patterns unique to specific cancer stages, along with a new method for assessing statistical significance of pathways enriched by co-expressed genes. Our test results have shown that our staging results are comparable with (or superior to) manual staging results by human pathologists. Highly exciting new insights are gained through our analyses of new pathways in cancer vs. controls as well as pathways that disappear gradually throughout the evolution of individual cancer types. We anticipate that the co-expression based analyses will prove to be an important direction for functional studies in cancer research.

## Data and methods

### Data

14 cancer types were initially selected since this set of cancers has been used in our previous studies^34–37^ as they each have sufficiently large number of samples in TCGA, namely: BLCA, BRCA, COAD, ESCA, HNSC, KICH, KIRC, KIRP, LIHC, LUAD, LUSC, PRAD, STAD, and THCA. Here, we further require that each cancer type have at least ten samples for each stage, which leaves only eight cancer types: BRCA, COAD, HNSC, KIRC, KIRP, LUAD, STAD, THCA. Table 7 gives the detailed information for each of the eight cancer types.

**Table 7:** The number of tissue samples for eight cancer types.

Cancer type	Control	Stage 1	Sage 2	Stage 3	Stage 4
BRCA	113	182	624	249	20
COAD	41	75	179	131	64
HNSC	44	25	70	78	261
KIRC	72	266	57	123	82
KIRP	32	172	22	51	15
LUAD	59	278	121	84	26
STAD	32	53	111	150	38
THCA	58	286	52	113	57

### Calculation of co-expressed genes

For a given set of cancer tissues and their transcriptomic data, we calculate the Pearson correlation coefficient $(\uprho )$ between each pair of expressed genes across the samples as follows:

$$\uprho \left(\mathrm{X},\mathrm{Y}\right)=\frac{\mathrm{E}\left(\mathrm{X}\mathrm{Y}\right)-\mathrm{E}(\mathrm{X})\mathrm{E}(\mathrm{Y})}{\sqrt{E({X}^{2}-{E}^{2}(X))}\sqrt{E({Y}^{2}-{E}^{2}(Y))}}$$

where *E*(X) is the expected value of expression levels of gene x across all samples. A pair of genes is deemed to be co-expressed if $\left|\uprho \left(\mathrm{X},\mathrm{Y}\right)\right|>0.7$ with * p* value < 0.05, where the * p* value is calculated as follows:

$$\mathrm{t}=\frac{\rho \times \sqrt{n-2}}{\sqrt{1-{\rho }^{2}}}$$

with n being the number of samples.

### Pathway enrichment

We have developed a new scoring scheme to assess the statistical significance of a pathway enriched by a set of co-expressed DEGs at a specific stage of a cancer type. For a pathway with *n* gene pairs containing *k* co-expressed gene pairs over a given set of cancer samples, the following hypergeometric distribution^38^ is used to calculate the statistical significance of this pathway enriched by the k gene pairs where N gene pairs are differentially expressed in cancer vs. controls, of which K pairs of genes are co-expressed:

$$\mathrm{P}\left(\mathrm{X}=\mathrm{k}\right)=\frac{\left(\genfrac{}{}{0pt}{}{K}{k}\right)\left(\genfrac{}{}{0pt}{}{N-K}{n-k}\right)}{\left(\genfrac{}{}{0pt}{}{N}{n}\right)}$$

## Supplementary information

Supplementary file1

Supplementary file2

Supplementary file3

Supplementary file4

Supplementary file5

Supplementary file6

Supplementary file7

Supplementary file8

Supplementary file9

Supplementary file10

## Data Availability

The data used to support the findings of this study are openly available from TCGA database (https://www.cancer.gov/about-nci/organization/ccg/research/structural-genomics/tcga).
