# Supplementary material for: Co-expression based cancer staging and application
Source: Sci Rep. 2020 Jun 30;10:10624. doi: 10.1038/s41598-020-67476-7 (PMC7327081; doi:10.1038/s41598-020-67476-7)
Supplement: Supplementary file 2 — Supplementary file2 [file 41598_2020_67476_MOESM2_ESM.docx]

Co-Expression based Cancer Staging and Application

Xiangchun Yu^2,3,5^, Sha Cao^4^, Yi Zhou^3^, Zhezhou Yu^2*^, and Ying Xu^1, 3*^

^1^Cancer Systems Biology Center, The China-Japan Union Hospital, and ^2^College of Computer Science and Technology, Jilin University, Changchun, China; ^3^Computational Systems Biology Lab, Department of Biochemistry and Molecular Biology and Institute of Bioinformatics, University of Georgia, Georgia, USA; ^4^Department of Biostatistics, Indiana University School of Medicine, Indianapolis, USA; and ^5^School of Information Engineering, Jiangxi University of Science and Technology, Ganzhou, China.

Correspondence authors: yuzz@jlu.edu.cn; [xyn@uga.edu](mailto:xyn@uga.edu).

The prediction results by other methods are summarized in Supplementary Tables S1(1-5). We note that most of the machine learning methods give comparable results except for Naive Bayes and random Ferns, whose performance is poorer than the others as detailed in the Tables S1(1-5).

**Table S1(1):** Prediction performance of cancer stages using the Naive Bayes method.

| **Stage** | **Measure** | **BRCA** | **COAD** | **HNSC** | **KIRC** | **KIRP** | **LUAD** | **STAD** | **THCA** |
| --- | --- | --- | --- | --- | --- | --- | --- | --- | --- |
| **1** | **Sensitivity** | 0.1778 | 0.6273 | 0.6143 | 0.7987 | 0.9745 | 0.8386 | 0.2 | 0.8388 |
|  | **Specificity** | 0.9962 | 0.9908 | 0.8942 | 0.8921 | 0.7 | 0.5909 | 0.9506 | 0.8906 |
| **2** | **Sensitivity** | 0.9317 | 0.8887 | 0.1 | 0.3938 | 0.2333 | 0.3857 | 0.4656 | 0.5933 |
|  | **Specificity** | 0.2797 | 0.8115 | 0.9757 | 0.959 | 0.9942 | 0.8772 | 0.8257 | 0.9858 |
| **3** | **Sensitivity** | 0.1986 | 0.7132 | 0.1957 | 0.8167 | 0.35 | 0.3292 | 0.6818 | 0.8061 |
|  | **Specificity** | 0.9535 | 0.8849 | 0.9577 | 0.7832 | 0.9541 | 0.9304 | 0.619 | 0.7853 |
| **4** | **Sensitivity** | 0.58 | 0.5056 | 0.9052 | 0.4417 | 0.675 | 0.1714 | 0.4727 | 0.2688 |
|  | **Specificity** | 0.9672 | 0.9336 | 0.464 | 0.955 | 0.9169 | 0.95 | 0.8868 | 0.9692 |
| **All** | **Accuracy** | 0.6285 | 0.7412 | 0.6339 | 0.7058 | 0.7827 | 0.6188 | 0.5206 | 0.7456 |
|  | **Kappa** | 0.229 | 0.6235 | 0.2852 | 0.553 | 0.5166 | 0.3372 | 0.2835 | 0.5831 |

**Table S1(2):** Prediction performance of cancer stages using the treebag method.

| **Stage** | **Measure** | **BRCA** | **COAD** | **HNSC** | **KIRC** | **KIRP** | **LUAD** | **STAD** | **THCA** |
| --- | --- | --- | --- | --- | --- | --- | --- | --- | --- |
| **1** | **Sensitivity** | 0.8074 | 0.9318 | 0.8286 | 0.9291 | 0.9824 | 0.9506 | 0.3267 | 0.9459 |
|  | **Specificity** | 0.9928 | 0.9927 | 0.8567 | 0.9658 | 0.975 | 0.9818 | 0.9034 | 0.9188 |
| **2** | **Sensitivity** | 0.9656 | 0.9585 | 0.365 | 0.8812 | 0.65 | 0.7457 | 0.6625 | 0.9133 |
|  | **Specificity** | 0.9594 | 0.9731 | 0.9449 | 0.9525 | 0.9812 | 0.9833 | 0.9471 | 0.9687 |
| **3** | **Sensitivity** | 0.8162 | 0.9158 | 0.4478 | 0.9389 | 0.8857 | 0.7625 | 0.7205 | 0.8121 |
|  | **Specificity** | 0.9886 | 0.9806 | 0.9202 | 0.9908 | 0.9918 | 0.9784 | 0.8017 | 0.9595 |
| **4** | **Sensitivity** | 0.86 | 0.9667 | 0.8948 | 0.8833 | 0.95 | 0.6571 | 0.7727 | 0.6188 |
|  | **Specificity** | 0.9331 | 0.9752 | 0.934 | 0.9832 | 0.9662 | 0.8923 | 0.867 | 0.9692 |
| **All** | **Accuracy** | 0.9025 | 0.9427 | 0.7268 | 0.9194 | 0.936 | 0.8584 | 0.65 | 0.8779 |
|  | **Kappa** | 0.8355 | 0.9191 | 0.5473 | 0.8782 | 0.8717 | 0.777 | 0.5019 | 0.7983 |

**Table S1(3):** Prediction performance of cancer stages using the RF method.

| **Stage** | **Measure** | **BRCA** | **COAD** | **HNSC** | **KIRC** | **KIRP** | **LUAD** | **STAD** | **THCA** |
| --- | --- | --- | --- | --- | --- | --- | --- | --- | --- |
| **1** | **Sensitivity** | 0.6981 | 0.9591 | 0.7857 | 0.9633 | 0.9824 | 0.9614 | 0.1067 | 0.9741 |
|  | **Specificity** | 0.9992 | 0.9991 | 0.865 | 0.9776 | 0.9208 | 0.9621 | 0.9977 | 0.8953 |
| **2** | **Sensitivity** | 0.9785 | 0.9774 | 0.44 | 0.925 | 0.0667 | 0.7371 | 0.725 | 0.9467 |
|  | **Specificity** | 0.9451 | 0.9769 | 0.9785 | 0.9576 | 0.9899 | 0.9658 | 0.9271 | 0.9612 |
| **3** | **Sensitivity** | 0.8284 | 0.95 | 0.2913 | 0.9333 | 0.7929 | 0.4542 | 0.8159 | 0.7758 |
|  | **Specificity** | 0.9849 | 0.9903 | 0.9788 | 0.9941 | 0.9426 | 0.9928 | 0.7328 | 0.9466 |
| **4** | **Sensitivity** | 0.9 | 0.9889 | 0.9675 | 0.8917 | 1 | 0.8 | 0.8273 | 0.3875 |
|  | **Specificity** | 0.929 | 0.9876 | 0.784 | 0.9939 | 0.9535 | 0.862 | 0.8747 | 0.9842 |
| **All** | **Accuracy** | 0.895 | 0.9679 | 0.752 | 0.9413 | 0.8747 | 0.8195 | 0.6843 | 0.8644 |
|  | **Kappa** | 0.8202 | 0.9545 | 0.5486 | 0.9109 | 0.7415 | 0.7114 | 0.5299 | 0.7709 |

**Table S1(4):** Prediction performance of cancer stages using the RFerns method.

| **Stage** | **Measure** | **BRCA** | **COAD** | **HNSC** | **KIRC** | **KIRP** | **LUAD** | **STAD** | **THCA** |
| --- | --- | --- | --- | --- | --- | --- | --- | --- | --- |
| **1** | **Sensitivity** | 0.7315 | 0.7409 | 0.3857 | 0.8114 | 0.049 | 0.2964 | 0.7133 | 0.8024 |
|  | **Specificity** | 0.4287 | 0.9394 | 0.9908 | 0.9513 | 1 | 0.9318 | 0.5759 | 0.9078 |
| **2** | **Sensitivity** | 0.1446 | 0.7981 | 0.64 | 0.5812 | 0.9333 | 0.3686 | 0.5594 | 0.5467 |
|  | **Specificity** | 0.9609 | 0.9128 | 0.7243 | 0.9993 | 0.0913 | 0.8912 | 0.9229 | 0.9993 |
| **3** | **Sensitivity** | 0.4135 | 0.7868 | 0.6696 | 0.8361 | 0.0857 | 0.875 | 0.4727 | 0.6273 |
|  | **Specificity** | 0.7576 | 0.8806 | 0.5798 | 0.9361 | 0.9508 | 0.4152 | 0.9 | 0.8922 |
| **4** | **Sensitivity** | 0.32 | 0.6667 | 0.2675 | 0.9042 | 0 | 0.0143 | 0.2636 | 0.7062 |
|  | **Specificity** | 0.986 | 0.9469 | 0.976 | 0.8595 | 1 | 0.9972 | 0.9824 | 0.8338 |
| **All** | **Accuracy** | 0.3091 | 0.7672 | 0.4055 | 0.8077 | 0.124 | 0.3933 | 0.5127 | 0.7275 |
|  | **Kappa** | 0.1114 | 0.6728 | 0.2353 | 0.7154 | 0.0273 | 0.1967 | 0.3459 | 0.5737 |

**Table S1(5):** Prediction performance of cancer stages using the WSRF method.

| **Stage** | **Measure** | **BRCA** | **COAD** | **HNSC** | **KIRC** | **KIRP** | **LUAD** | **STAD** | **THCA** |
| --- | --- | --- | --- | --- | --- | --- | --- | --- | --- |
| **1** | **Sensitivity** | 0.463 | 0.9409 | 0.9 | 0.9468 | 0.9784 | 0.9602 | 0.1933 | 0.9741 |
|  | **Specificity** | 0.9811 | 0.9908 | 0.8633 | 0.9632 | 0.8542 | 0.8318 | 0.9759 | 0.8562 |
| **2** | **Sensitivity** | 0.9737 | 0.9585 | 0.435 | 0.8938 | 0.0833 | 0.6829 | 0.6969 | 0.96 |
|  | **Specificity** | 0.8263 | 0.9654 | 0.9636 | 0.9655 | 1 | 0.9649 | 0.9229 | 0.9799 |
| **3** | **Sensitivity** | 0.7797 | 0.9316 | 0.3696 | 0.9611 | 0.6786 | 0.4167 | 0.825 | 0.8061 |
|  | **Specificity** | 0.9796 | 0.9785 | 0.9808 | 0.9882 | 0.9656 | 0.9888 | 0.7069 | 0.9422 |
| **4** | **Sensitivity** | 0.92 | 0.9778 | 0.9701 | 0.8875 | 0.925 | 0.6286 | 0.6818 | 0.3062 |
|  | **Specificity** | 0.9443 | 0.9929 | 0.87 | 0.9924 | 0.9183 | 0.8979 | 0.9066 | 0.9872 |
| **All** | **Accuracy** | 0.8414 | 0.9504 | 0.7732 | 0.9355 | 0.848 | 0.7919 | 0.6765 | 0.8638 |
|  | **Kappa** | 0.7147 | 0.9295 | 0.6011 | 0.9018 | 0.6814 | 0.6417 | 0.5142 | 0.7657 |
