# Supplementary material for: Co-expression based cancer staging and application
Source: Sci Rep. 2020 Jun 30;10:10624. doi: 10.1038/s41598-020-67476-7 (PMC7327081; doi:10.1038/s41598-020-67476-7)
Supplement: Supplementary file 3 — Supplementary file3 [file 41598_2020_67476_MOESM3_ESM.docx]

Co-Expression based Cancer Staging and Application

Xiangchun Yu^2,3,5^, Sha Cao^4^, Yi Zhou^3^, Zhezhou Yu^2*^, and Ying Xu^1, 3*^

^1^Cancer Systems Biology Center, The China-Japan Union Hospital, and ^2^College of Computer Science and Technology, Jilin University, Changchun, China; ^3^Computational Systems Biology Lab, Department of Biochemistry and Molecular Biology and Institute of Bioinformatics, University of Georgia, Georgia, USA; ^4^Department of Biostatistics, Indiana University School of Medicine, Indianapolis, USA; and ^5^School of Information Engineering, Jiangxi University of Science and Technology, Ganzhou, China.

Correspondence authors: yuzz@jlu.edu.cn; [xyn@uga.edu](mailto:xyn@uga.edu).

To understand what might be the reasons for “incorrect” predictions by our method compared to the **annotated** stages in TCGA by pathologists, we have examined the prediction results for BRCA, COAD, KIRC, KIRP, LUAD, and THCA. The following Tables S2(1-6) list, for each stage, the numbers of samples correctly predicted and of predicted to earlier as well as later stages, respectively.

**Table S2(1)**: The confusion matrix for predicted vs. annotated stage of BRCA.

| **Predicted/ Annotated** | **stage 1** | **stage 2** | **stage 3** | **stage 4** |
| --- | --- | --- | --- | --- |
| **stage 1** | 52 | 5 | 0 | 0 |
| **stage 2** | 1 | 180 | 5 | 0 |
| **stage 3** | 0 | 0 | 63 | 0 |
| **stage 4** | 1 | 1 | 6 | 5 |

**Table S2(2)**: The confusion matrix for predicted vs. annotated stage of COAD.

| **Predicted/ Annotated** | **stage 1** | **stage 2** | **stage 3** | **stage 4** |
| --- | --- | --- | --- | --- |
| **stage 1** | 22 | 1 | 0 | 0 |
| **stage 2** | 0 | 49 | 1 | 0 |
| **stage 3** | 0 | 1 | 35 | 0 |
| **stage 4** | 0 | 2 | 2 | 18 |

**Table S2(3)**: The confusion matrix for predicted vs. annotated stage of KIRC.

| **Predicted/ Annotated** | **stage 1** | **stage 2** | **stage 3** | **stage 4** |
| --- | --- | --- | --- | --- |
| **stage 1** | 75 | 0 | 0 | 5 |
| **stage 2** | 4 | 16 | 1 | 0 |
| **stage 3** | 0 | 0 | 35 | 0 |
| **stage 4** | 0 | 0 | 0 | 19 |

**Table S2(4)**: The confusion matrix for predicted vs. annotated stage of KIRP.

| **Predicted/ Annotated** | **stage 1** | **stage 2** | **stage 3** | **stage 4** |
| --- | --- | --- | --- | --- |
| **stage 1** | 42 | 0 | 0 | 0 |
| **stage 2** | 0 | 2 | 0 | 0 |
| **stage 3** | 9 | 1 | 14 | 0 |
| **stage 4** | 0 | 3 | 0 | 4 |

**Table S2(5)**: The confusion matrix for predicted vs. annotated stage of LUAD.

| **Predicted/ Annotated** | **stage 1** | **stage 2** | **stage 3** | **stage 4** |
| --- | --- | --- | --- | --- |
| **stage 1** | 70 | 0 | 0 | 0 |
| **stage 2** | 6 | 30 | 3 | 1 |
| **stage 3** | 3 | 4 | 13 | 2 |
| **stage 4** | 4 | 1 | 8 | 4 |

**Table S2(6)**: The confusion matrix for predicted vs. annotated stage of THCA.

| **Predicted/ Annotated** | **stage 1** | **stage 2** | **stage 3** | **stage 4** |
| --- | --- | --- | --- | --- |
| **stage 1** | 84 | 0 | 3 | 6 |
| **stage 2** | 1 | 12 | 2 | 1 |
| **stage 3** | 0 | 0 | 24 | 6 |
| **stage 4** | 0 | 3 | 4 | 3 |
