# Supplementary material for: Co-expression based cancer staging and application
Source: Sci Rep. 2020 Jun 30;10:10624. doi: 10.1038/s41598-020-67476-7 (PMC7327081; doi:10.1038/s41598-020-67476-7)
Supplement: Supplementary file 4 — Supplementary file4 [file 41598_2020_67476_MOESM4_ESM.docx]

Co-Expression based Cancer Staging and Application

Xiangchun Yu^2,3,5^, Sha Cao^4^, Yi Zhou^3^, Zhezhou Yu^2*^, and Ying Xu^1, 3*^

^1^Cancer Systems Biology Center, The China-Japan Union Hospital, and ^2^College of Computer Science and Technology, Jilin University, Changchun, China; ^3^Computational Systems Biology Lab, Department of Biochemistry and Molecular Biology and Institute of Bioinformatics, University of Georgia, Georgia, USA; ^4^Department of Biostatistics, Indiana University School of Medicine, Indianapolis, USA; and ^5^School of Information Engineering, Jiangxi University of Science and Technology, Ganzhou, China.

Correspondence authors: yuzz@jlu.edu.cn; [xyn@uga.edu](mailto:xyn@uga.edu).

To understand what might be the reasons for “incorrect” predictions by our method compared to the **annotated** stages in TCGA by pathologists, we have examined the prediction results for BRCA, COAD, KIRC, KIRP, LUAD, and THCA. The following Figures S1(1-6) show the distributions of the numbers of DEGs in stage-specific samples for predicted (a) and annotated (b) stages of the six cancer types, respectively.


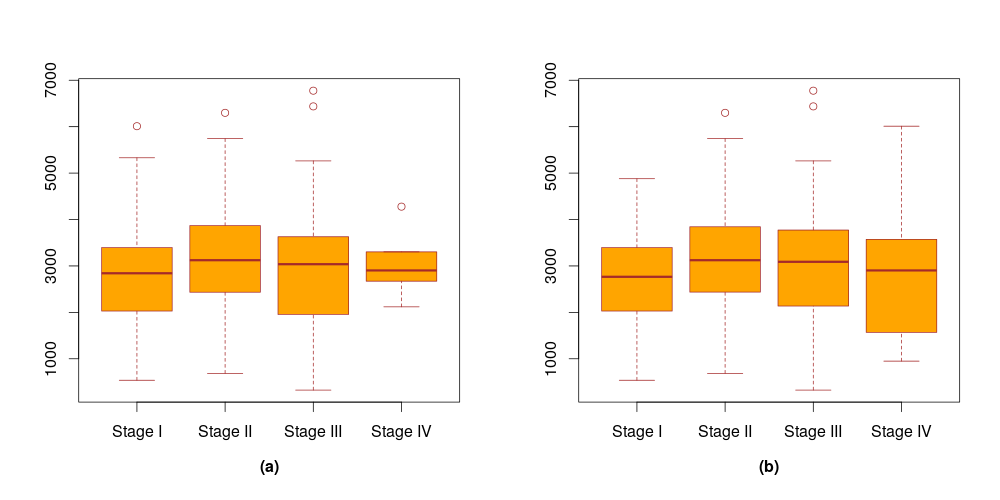


**Figure S1(1):** The numbers of DEGs in stage-specific samples for annotated (a) and predicted (b) stages of BRCA.


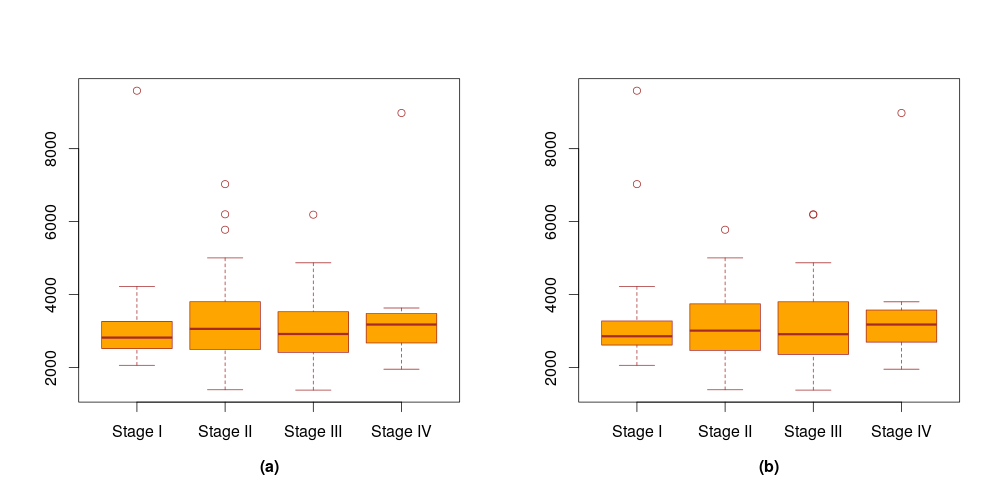


**Figure S1(2):** The numbers of DEGs in stage-specific samples for annotated (a) and predicted (b) stages of COAD.


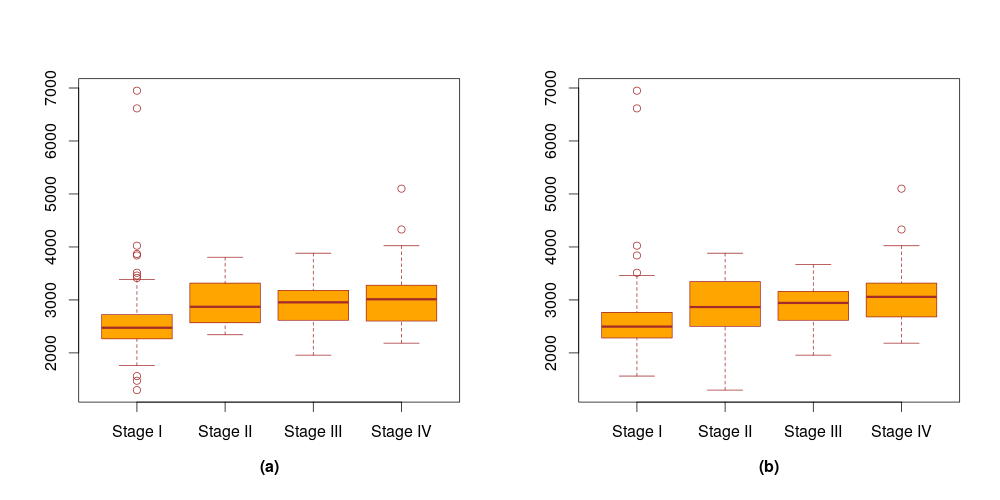


**Figure S1(3):** The numbers of DEGs in stage-specific samples for annotated (a) and predicted (b) stages of KIRC.


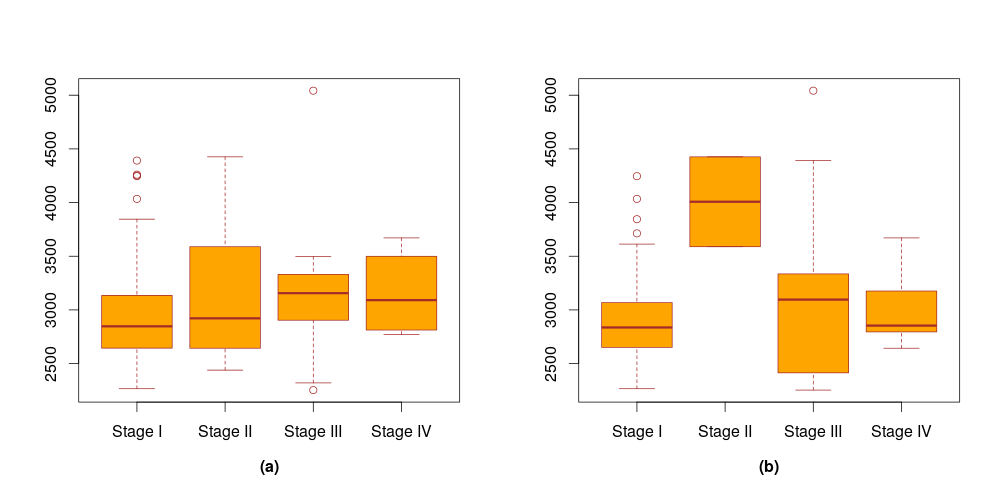


**Figure S1(4):** The numbers of DEGs in stage-specific samples for annotated (a) and predicted (b) stages of KIRP.


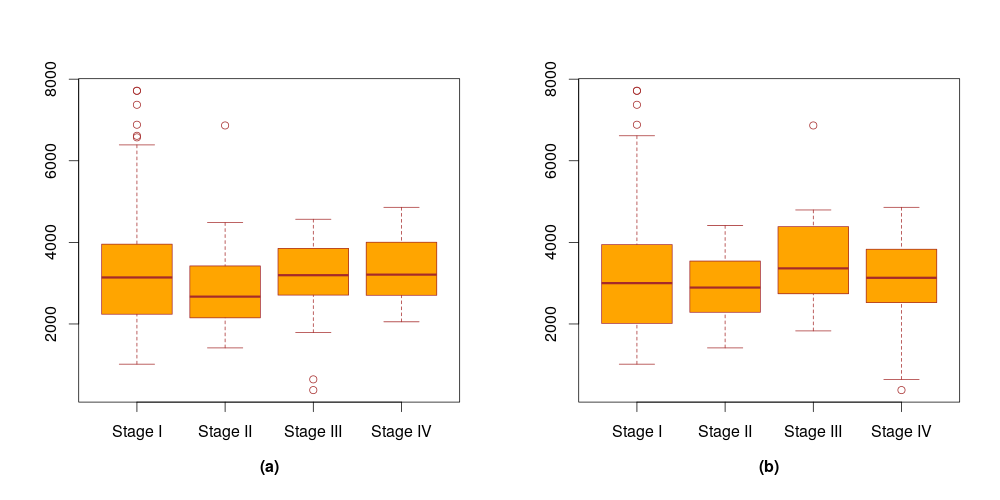


**Figure S1(5):** The numbers of DEGs in stage-specific samples for annotated (a) and predicted (b) stages of LUAD.


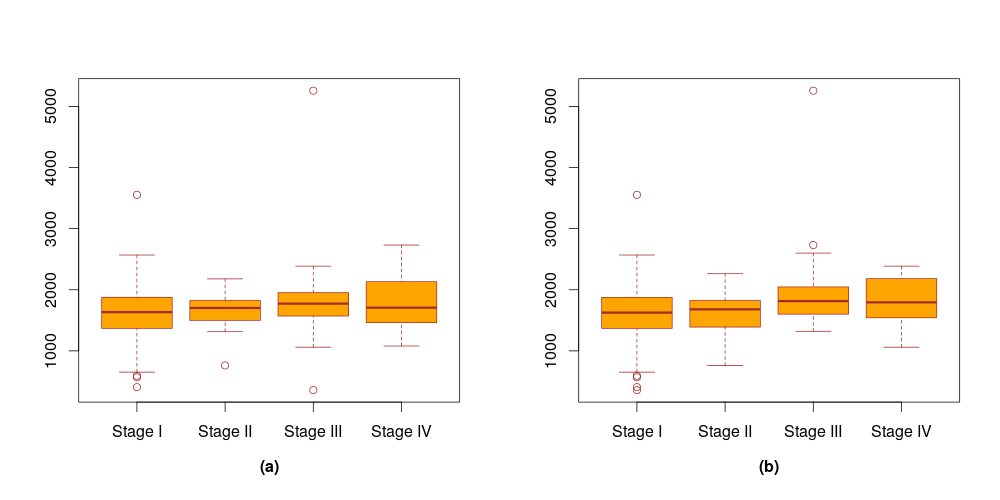


**Figure S1(6):** The numbers of DEGs in stage-specific samples for annotated (a) and predicted (b) stages of THCA.
